# Myeloid cell-specific ablation of *Runx2* gene exacerbates post-infarct cardiac remodeling

**DOI:** 10.1038/s41598-022-21202-7

**Published:** 2022-10-05

**Authors:** Masashi Tomimatsu, Kotaro Matsumoto, Moe Ashizuka, Shohei Kumagai, Shota Tanaka, Takafumi Nakae, Kosei Yokota, Shunsuke Kominami, Ryota Kajiura, Daisuke Okuzaki, Daisuke Motooka, Aki Shiraishi, Takaya Abe, Hideo Matsuda, Yoshiaki Okada, Makiko Maeda, Shigeto Seno, Masanori Obana, Yasushi Fujio

**Affiliations:** 1 Laboratory of Clinical Science and Biomedicine, Graduate School of Pharmaceutical Sciences, Osaka University, Suita, Osaka Japan; 2 Genome Information Research Center, Research Institute for Microbial Diseases, Osaka University, Suita, Osaka Japan; 3 Laboratory for Animal Resources and Genetic Engineering, RIKEN Center for Biosystems Dynamics Research, Kobe, Japan; 4 Department of Bioinformatic Engineering, Graduate School of Information Science and Technology, Osaka University, Suita, Osaka Japan; 5 Laboratory of Clinical Pharmacology, Graduate School of Pharmaceutical Sciences, Osaka University, Suita, Osaka Japan; 6 Medical Center for Translational Research, Department of Medical Innovation, Osaka University Hospital, Suita, Osaka Japan; 7 Integrated Frontier Research for Medical Science Division, Institute for Open and Transdisciplinary Research Initiatives, Osaka University, Suita, Japan; 8 Global Center for Medical Engineering and Informatics (MEI), Osaka University, Suita, Osaka Japan; 9 Radioisotope Research Center, Institute for Radiation Science, Osaka University, Suita, Osaka Japan

**Keywords:** Cell biology, Cardiology

## Abstract

Runt-related transcription factor 2 (Runx2), a regulator of osteoblast differentiation, is pathologically involved in vascular calcification; however, the significance of Runx2 in cardiac homeostasis remains unclear. Here, we investigated the roles of Runx2 in cardiac remodeling after myocardial infarction (MI). The expression of Runx2 mRNA and protein was upregulated in murine hearts after MI. Runx2 was expressed in heart-infiltrating myeloid cells, especially in macrophages, at the border zone of post-infarct myocardium. To analyze the biological functions of Runx2 in cardiac remodeling, myeloid cell-specific *Runx2* deficient (CKO) mice were exposed to MI. After MI, ventricular weight/tibia length ratio was increased in CKO mice, concomitant with severe cardiac dysfunction. Cardiac fibrosis was exacerbated in CKO mice, consistent with the upregulation of collagen 1a1 expression. Mechanistically, immunohistochemical analysis using anti-CD31 antibody showed that capillary density was decreased in CKO mice. Additionally, conditioned culture media of myeloid cells from Runx2 deficient mice exposed to MI induced the tube formation of vascular endothelial cells to a lesser extent than those from control mice. RNA-sequence showed that the expression of pro-angiogenic or anti-angiogenic factors was altered in macrophages from Runx2-deficient mice. Collectively, *Runx2*^+^ myeloid cells infiltrate into post-infarct myocardium and prevent adverse cardiac remodeling, at least partially, by regulating endothelial cell function.

## Introduction

Myocardial infarction (MI) is one of the leading causes of heart failure (HF). Following ischemia and/or reperfusion injury at the acute phase, cardiomyocyte loss and fibrosis are observed during the subacute to the chronic phase, known as adverse cardiac remodeling^1–4^. Since neurohumoral factors, such as adrenergic signals and renin–angiotensin–aldosterone system, are involved in cardiac remodeling, blockade of these factors is clinically recommended as pharmacotherapies against HF^5,6^. Recently, much attention has been paid to the inflammation as a critical event for cardiac remodeling. As a result, it has been revealed that various kinds of inflammatory cells play important roles in the progression/prevention of post-infarct cardiac remodeling^7–11^, suggesting that the modulation of inflammatory signaling pathways could be a novel therapeutic strategy.

Immediately after MI, neutrophils infiltrate into the heart and evoke the inflammation, followed by the infiltration of macrophages. So far, the M1/M2 classification has been applied to heart-infiltrating macrophages. M1 macrophages produce pro-inflammatory cytokines and exacerbate myocardial inflammation at an early stage^12^. Afterwards, macrophages are phenotypically modulated into M2 macrophages and produce anti-inflammatory cytokines and growth factors, contributing to wound healing^13^. Importantly, accumulating evidence has demonstrated that heart-infiltrating macrophages cannot be classified simply into conventional subsets according to M1/M2 theory. Therefore, the characterization of myeloid cells by detailed analyses of gene expression profile is essential to understand the mechanism of cardiac remodeling.

Recent advances in osteoimmunology have demonstrated that bone-related genes are closely associated with inflammation. For example, osteopontin, which was originally identified as an adhesion molecule in bone tissue^14^, is related with cardiac inflammation in experimental animal models and patients with dilated cardiomyopathy^15–17^. Runt-related transcription factor 2 (Runx2) is a master gene responsible for osteoblast differentiation and bone formation^18^. Interestingly, Runx2 is expressed not only in osteoblasts but also in a wide range of cell-lineages. In cardiovascular diseases, pathological expression of Runx2 results in vascular calcification by promoting differentiation of macrophages into osteoclast-like cells^19^. However, the pathophysiological significance of Runx2 in cardiac remodeling remains to be fully elucidated.

In the present study, to clarify the biological significance of Runx2 in cardiac remodeling after MI, we employed murine coronary ligation model. The expression of Runx2 mRNA and protein was upregulated in post-infarct myocardium. Runx2 was expressed in heart-infiltrating CD11b^+^ myeloid cells, including macrophages. Myeloid cell-specific *Runx2* gene ablation exacerbated cardiac function and fibrosis, associated with reduced capillary density, after MI. Consistently, tube formation assay showed that conditioned culture media of myeloid cells from *Runx2* deficient mice exposed to MI exhibited reduced angiogenic activity. Moreover, myeloid cell-specific *Runx2* gene ablation affected the production of pro-/anti-angiogenic factors in macrophages. This is the first demonstration that myeloid Runx2 is a critical regulator that prevents cardiac remodeling in post-infarct myocardium.

## Results

### Runx2 is expressed in myeloid cells that infiltrate into post-infarct myocardium

**Figure 1:**
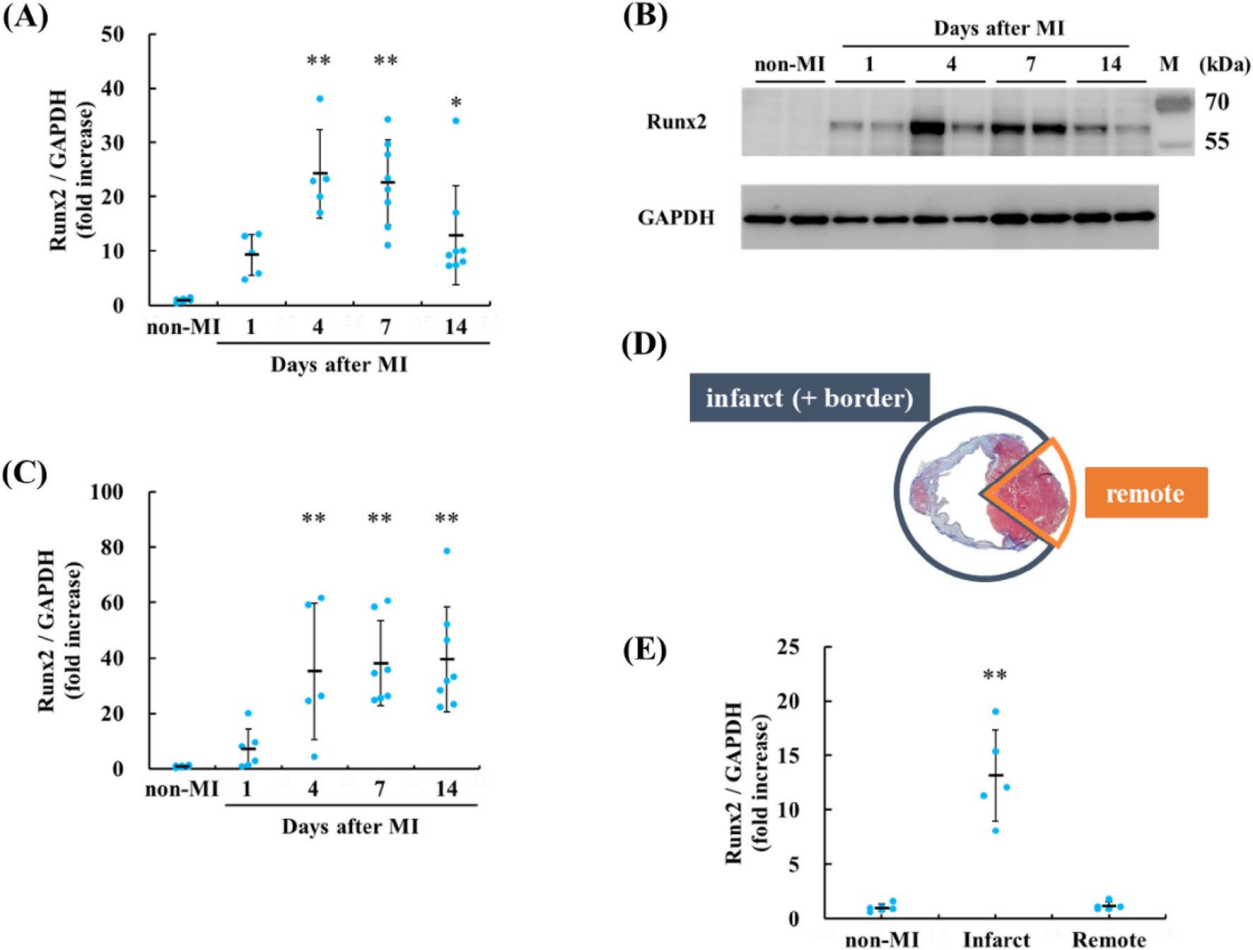
The expression of Runx2 was upregulated in the murine hearts after MI. (**A**) mRNA expression of Runx2 was analyzed at indicated time-points by quantitative RT-PCR. The results were normalized to that of GAPDH. Data are shown as mean ± SD (n = 5 mice for MI1d, 4d, n = 6 mice for non-MI, n = 8 mice for MI7d, 14d), *P < 0.05, **P < 0.01 vs non-MI by Kruskal–Wallis test followed by Steel test. (**B**) The heart lysates from WT mice at 1, 4, 7, and 14 days after MI were immunoblotted with anti-Runx2 and anti-GAPDH antibodies. The heart lysates from non-MI mice were used as a control. Representative images are shown. (**C**) Runx2 expression was quantified. Data are shown as mean ± SD (n = 5 mice for MI4d, n = 6 mice for non-MI, MI1d, n = 7 mice for MI7d, n = 8 mice for MI14d), **P < 0.01 vs non-MI by Kruskal–Wallis test followed by Steel test. (**D**) Masson’s trichrome staining was performed to identify the infarct (+ border) and the remote areas. Representative image is shown. (**E**) The mRNA expression of Runx2 was quantified 7 days after MI by quantitative RT-PCR. Data are shown as mean ± SD (n = 5 mice for MI7d, n = 6 mice for non-MI), **P < 0.01 vs non-MI, remote area by Kruskal–Wallis test following by Steel–Dwass test.

To address whether Runx2 is involved in cardiac remodeling, the cardiac expression of Runx2 mRNA was evaluated after MI by quantitative RT-PCR (Fig. 1A). The expression of Runx2 mRNA peaked 4 to 7 days after MI and gradually declined. Consistently, immunoblot analyses with anti-Runx2 antibody revealed that the expression of Runx2 protein was upregulated after MI (Fig. 1B,C). Next, we divided the infarcted hearts into two areas after MI, infarct area and remote area, and analyzed the expression of Runx2 by qRT-PCR (Fig. 1D,E). Runx2 mRNA was highly expressed in infarct area, compared with remote area.

**Figure 2:**
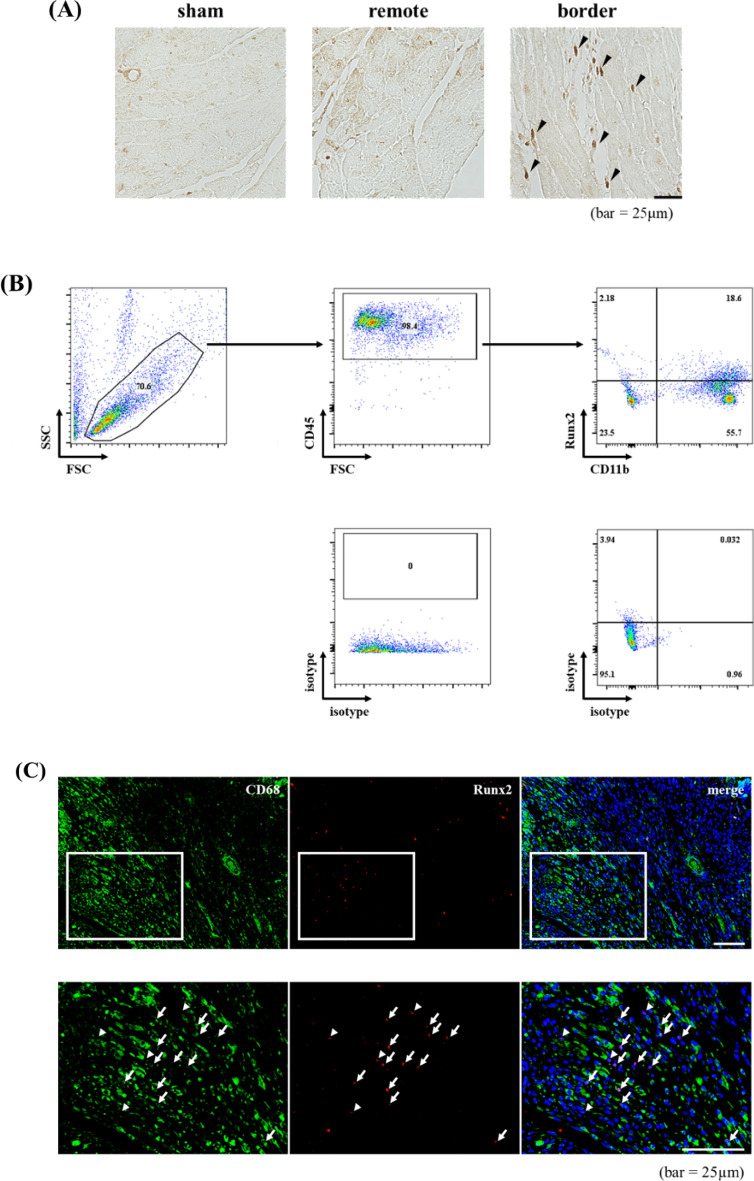

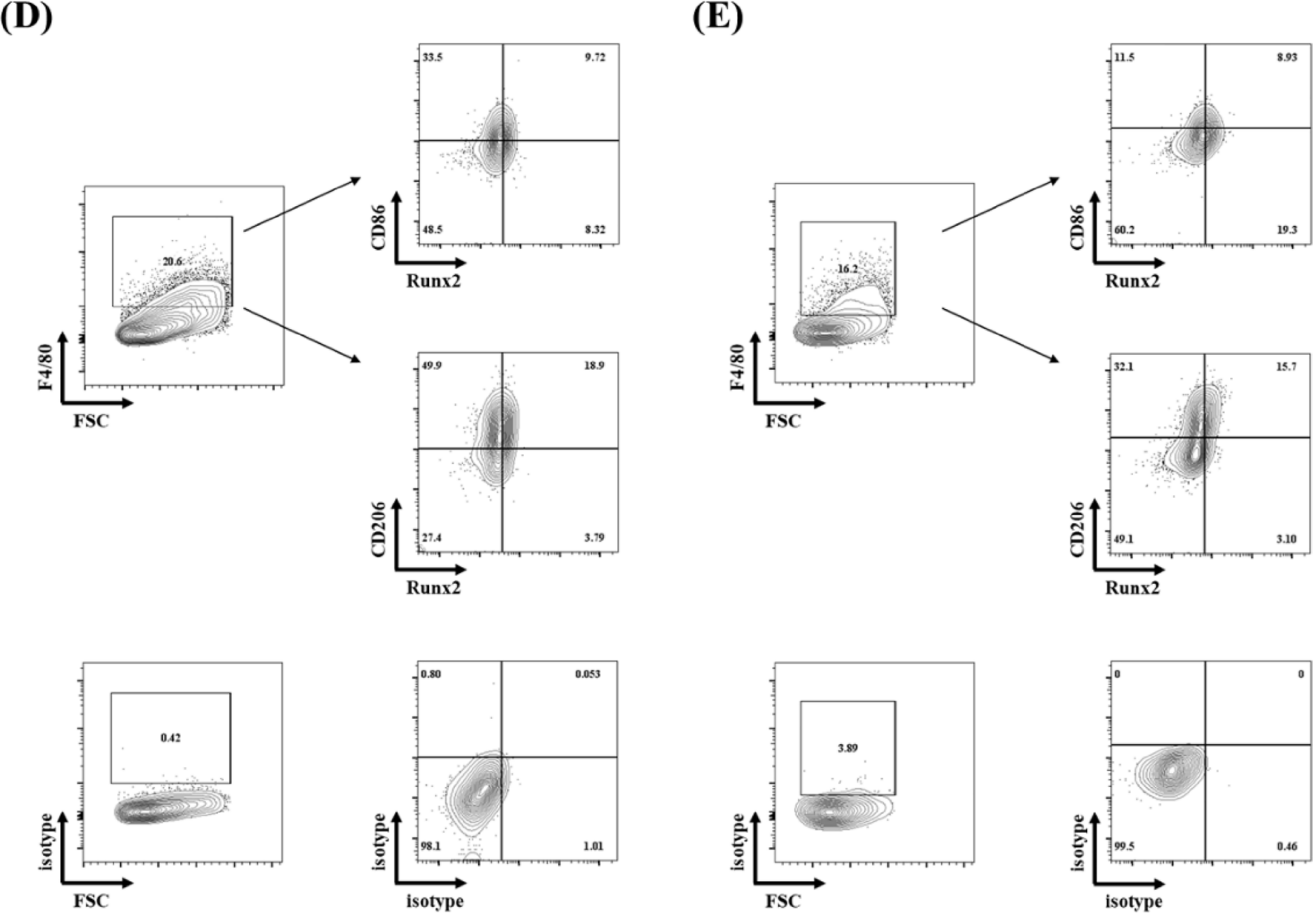
Runx2 was expressed in heart-infiltrating myeloid cells. (**A**) Paraffin-embedded sections of infarcted myocardium were prepared from the heart 7 days after MI. The sections were stained with anti-Runx2 antibody. Representative images are shown. (bar = 25 μm). (**B**) Representative flow cytometry plot of leukocytes enriched from MI7d hearts. (**C**) Frozen sections were prepared from the hearts 7 days after MI. The sections were co-stained with anti-Runx2 and anti-CD68 antibodies. Representative images are shown. Arrows, Runx2^+^ CD68^+^ cells: arrow heads, Runx2^+^ CD68^-^ cells. (bar = 50 μm). (**D** and **E**) Flow cytometry analysis of Runx2 expression on F4/80^+^ CD86^+^ M1 macrophages (upper) and F4/80^+^ CD206^+^ M2 macrophages (lower). CD11b + myeloid cells were enriched from MI4d (**D**) or MI7d (**E**) hearts. Isotype control antibodies were used for positive/negative gate setting.

Consistently, immunohistochemical analysis revealed that the infiltrating cells were positively stained with anti-Runx2 antibody at the border zone of post-infarct myocardium (Fig. 2A). Since the infiltrating cells mainly consist of myeloid cells, we examined whether heart-infiltrated myeloid cells express Runx2 by FACS analysis. More than 70% of CD45^+^ infiltrating cells were positively stained with anti-CD11b antibody (myeloid marker) 7 days after MI. Importantly, about 20% of CD11b were positively stained with anti-Runx2 antibody (Fig. 2B). As macrophages are major myeloid cells that infiltrate into myocardium 4–7 days after MI^20^, the heart sections were co-stained with anti-Runx2 and anti-CD68 (macrophage marker) antibodies (Fig. 2C). Importantly, a large population of Runx2-poistive cells were also stained with anti-CD68 antibody. Since Runx2^+^ CD68^-^ cells were also observed in border and infarct area of infarcted myocardium, where fibrosis develops, Runx2 expression in myofibroblasts was investigated (Supplementary Fig. S1). Immunofluorescence staining with anti-Runx2 and anti-periostin (myofibroblast marker) antibodies demonstrated that Runx2 was also expressed in myofibroblasts; however, since the ratio of Runx2^+^periostin^+^ cells to Runx2^+^ cells was small, we focused on Runx2 which is expressed in myeloid cells. Since macrophages are classified into M1 and M2 subsets, we examined which subset expresses Runx2 using the antibodies against CD86 or CD206, M1 or M2 marker, respectively (Fig. 2D,E) and found that both M1 and M2 macrophages expressed Runx2.

### Myeloid cell-specific Runx2 ablation deteriorated adverse cardiac remodeling after MI

**Figure 3:**
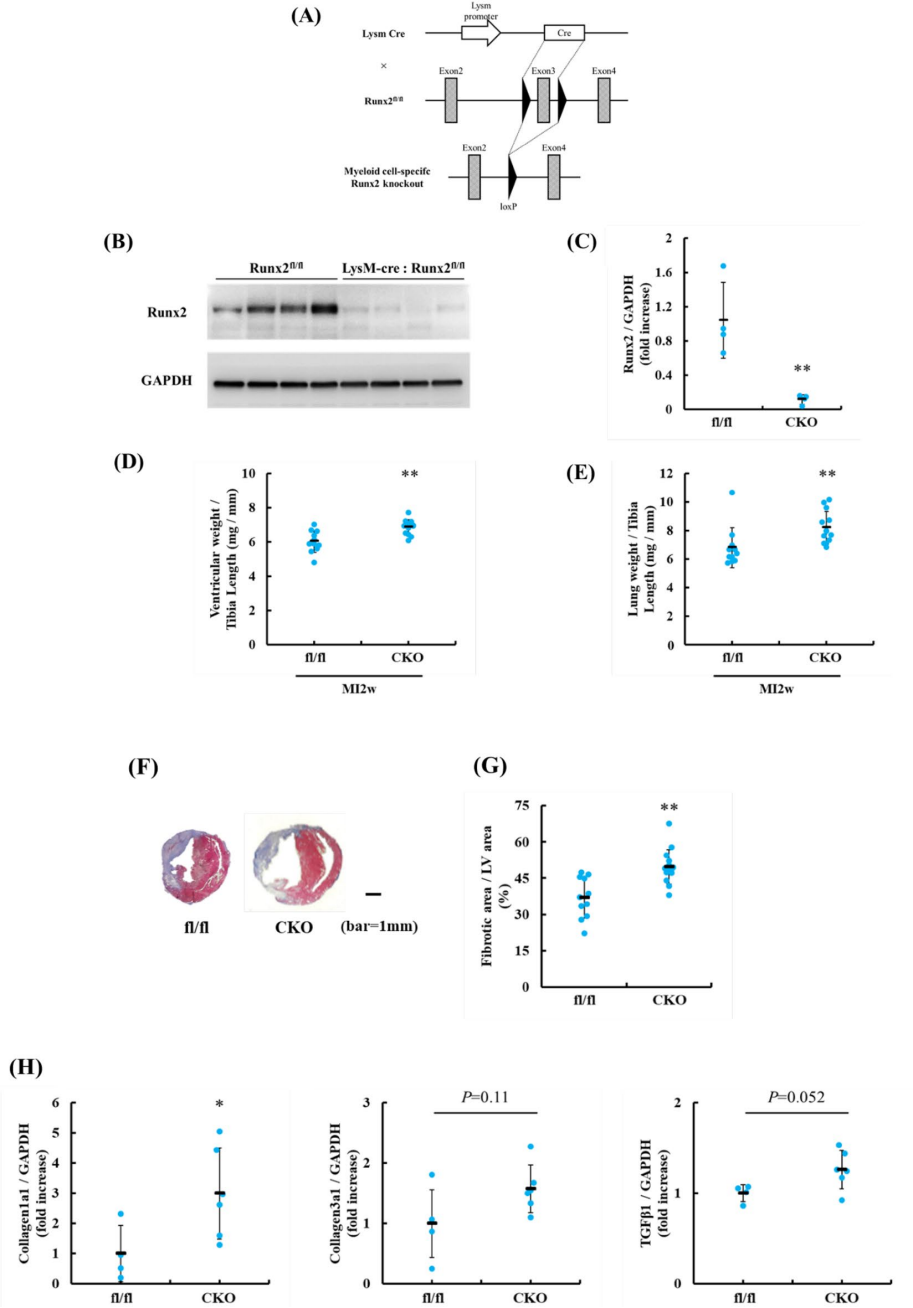
Myeloid cell-specific Runx2 ablation deteriorated adverse cardiac remodeling after MI. (**A**) Simplified scheme using Cre-loxP system is shown to generate myeloid cell-specific Runx2-deficient mice. (**B**, **C**) Seven days after MI, the sorted CD11b^+^ F4/80^+^ cells lysates from Runx2^fl/fl^, a control, and Runx2 CKO mice were immunoblotted with anti-Runx2 and anti-GAPDH antibodies. (**B**) Representative images are shown. (**C**) Runx2 expression was quantified. Data are shown mean ± SD (n = 4), **P < 0.01 vs Runx2^fl/fl^ by Welch’s *t*-test. (**D**, **E**) Ratios of ventricular weight to tibia length (**D**) and lung weight to tibia length (**E**) were calculated 14 days after MI. Data were shown as mean ± SD (n = 11 for Runx2^fl/fl^ mice, n = 13 for Runx2 CKO mice), *P < 0.05, **P < 0.01 vs Runx2^fl/fl^ by Mann Whitney’s *U* test. (**F**, **G**) Heart sections were prepared from Runx2^fl/fl^ and Runx2 CKO mice 14 days after MI and stained with Masson’s trichrome method. (**F**) Representative images are shown. (**G**) The ratio of fibrotic area to LV area was quantitatively estimated. Data are shown as mean ± SD (n = 11 for Runx2^fl/fl^ mice, n = 14 for Runx2 CKO mice), **P < 0.01 vs Runx2^fl/fl^ by Student’s *t*-test. (**H**) Quantitative RT-PCR was performed for *collagen1a1*, *collagen3*, and *Tgfβ1* 7 days after MI. The results were normalized to that of GAPDH. Data are shown mean ± SD (n = 4 for Runx2^fl/fl^ mice, n = 6 for Runx2 CKO mice). *P < 0.05 vs Runx2^fl/fl^ by Mann Whitney’s *U* test.

To determine the functional roles of Runx2 in cardiac function, myeloid cell-specific Runx2 conditional knockout (Runx2 CKO) mice were generated by crossbreeding Runx2^fl/fl^ mice with LysM-Cre mice. (Fig. 3A). Runx2^fl/fl^ mice were used as controls. CD11b^+^ F4/80^+^ macrophages were sorted from Runx2^fl/fl^ and Runx2 CKO hearts 7 days after MI, and the protein expression of Runx2 was assessed using immunoblotting. Runx2 was verified to be remarkably diminished in macrophages (Fig. 3B,C). To assess the effects of Runx2 ablation on the baseline phenotype, peripheral blood was collected from non-MI mice, and then, the number of various leukocyte subsets was measured. Myeloid cell-specific Runx2 ablation didn’t affected the number of neutrophils, monocytes, eosinophils and basophils, but reduced that of lymphocytes (Supplementary Fig. S2). Importantly, gravimetric analysis and echocardiographic showed that Runx2 depletion in myeloid cells didn’t affect baseline cardiac growth and function (Supplementary Fig. S3, Table1).

**Table 1:** Cardiac functions of Runx2^fl/fl^ and Runx2 CKO mice after MI.

	Day0		Day7		Day14	
	fl/fl	CKO	fl/fl	CKO	fl/fl	CKO
LVIDd (cm)	0.37 ± 0.02	0.37 ± 0.02	0.43 ± 0.03	0.47 ± 0.08	0.48 ± 0.02^††^	0.50 ± 0.05^††^
LVIDs (cm)	0.17 ± 0.01	0.17 ± 0.02	0.25 ± 0.02	0.30 ± 0.08	0.31 ± 0.01^††^	0.36 ± 0.03*^††^
FS (%)	54.0 ± 3.1	53.2 ± 2.8	41.9 ± 3.7	37.4 ± 5.1	34.7 ± 4.5^††^	29.0 ± 2.8*^††^

LVIDd = left ventricular internal dimension at diastole; LVIDs = left ventricular internal dimension at systole; FS = fractional shortening. Data are shown as mean ± SD (Runx2^fl/fl^ mice: n = 5, Runx2 CKO mice: n = 6). *P < 0.05 vs Runx2^fl/fl^ at Day14 by Student’s *t*-test, ^††^*P* < 0.01 vs Day0 by Dunnett test.

To elucidate the effects of Runx2 on the progression of cardiac remodeling, MI was generated in control and Runx2 CKO mice. Echocardiographic examination revealed that cardiac dysfunction was accelerated in Runx2 CKO mice 14 days after MI, compared with control mice (Table 1). Consistently, ventricular or lung weight normalized to tibia length was significantly increased in Runx2 CKO mice (Fig. 3D,E). Masson’s trichrome staining revealed that myeloid cell-specific *Runx2* ablation increased fibrotic area (Fig. 3F,G). Consistently, qPCR demonstrated that Runx2 CKO showed increased expression of *Collagen1a1* mRNA and that the expression of *Collagen3a1* and *TGFβ1* had tendency to be enhanced in CKO mice, though their increase was not statistically significant (Fig. 3H). We also assessed whether the Runx2 in macrophages have pro-fibrotic property by using bone marrow derived macrophages (BMDMs) and found that Runx2 ablation upregulated the expression of TGFβ1 in BMDMs (Supplementary Fig. S4). To examine the effects of myeloid-cell specific Runx2 deletion on cardiomyocyte death, terminal deoxynucleotidyl transferase-mediated dUTP nick-end labeling (TUNEL) staining was performed 7 days after MI. TUNEL staining revealed that Runx2 ablation in myeloid cells didn’t affect cardiomyocyte apoptosis after MI (Supplementary Fig. S5). Moreover, Runx2 CKO mice showed lower survival rates and tended to have higher rates of cardiac rupture compared with control mice (Supplementary Fig. S6). These data suggested that Runx2^+^ myeloid cells contribute to suppression of adverse cardiac remodeling, proposing the anti-fibrotic function of Runx2 in myeloid cells.

### Runx2^+^ myeloid cells were involved in post-infarct angiogenesis

**Figure 4:**
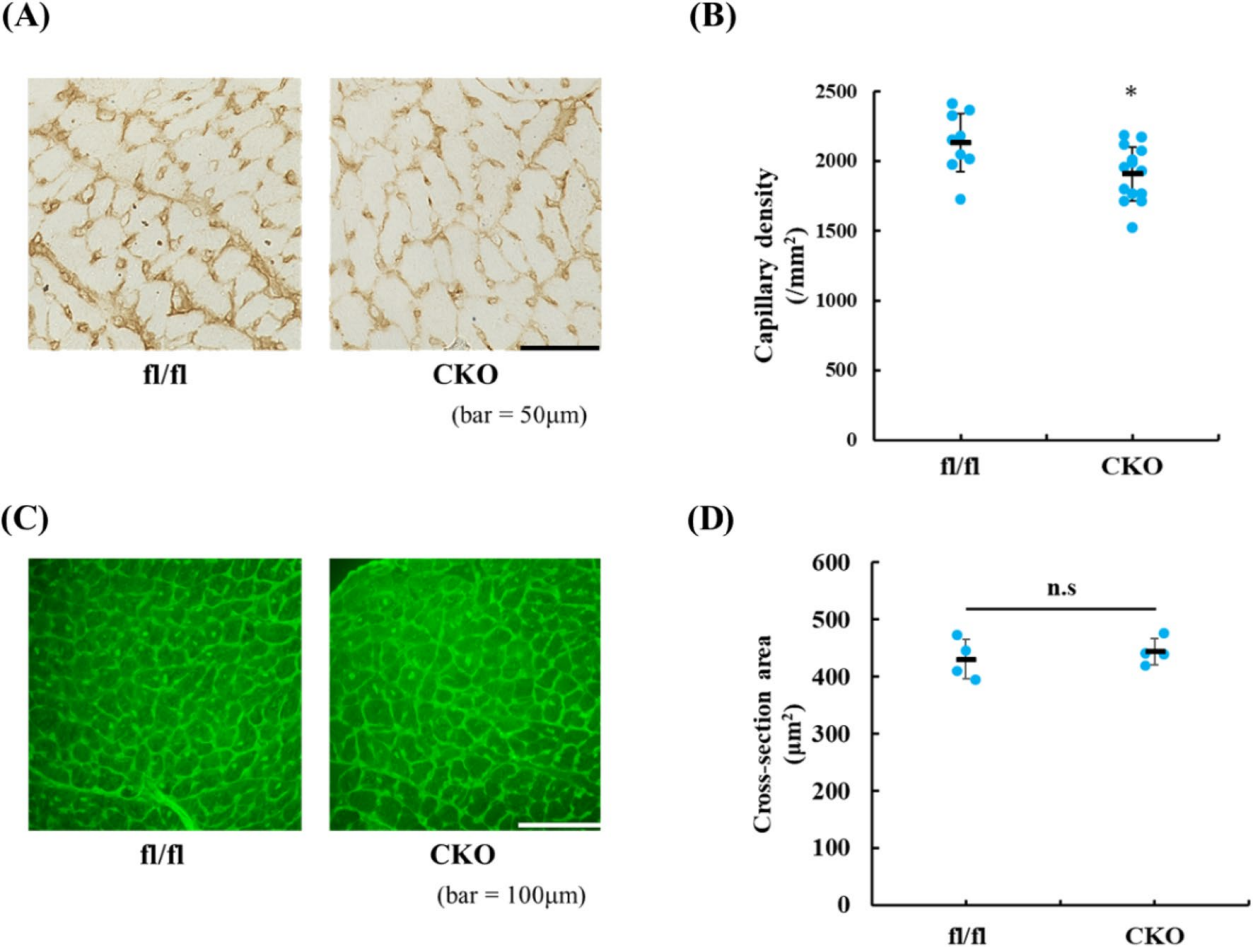
Capillary density was reduced in the post-infarct myocardium of Runx2 CKO mice. (**A**) Heart sections were prepared from Runx2^fl/fl^ and Runx2 CKO mice 14 days after MI. The sections were stained with anti-CD31 antibody. Representative images are shown. (**B**) Capillary density was quantitatively estimated. Data are shown as mean ± SD (n = 11 for Runx2^fl/fl^ mice, n = 14 for Runx2 CKO mice), *P < 0.05 vs Runx2^fl/fl^ by Student’s *t*-test. (**C**) Heart sections were prepared from Runx2^fl/fl^ and Runx2 CKO mice 14 days after MI. The sections were stained with wheat germ lectin conjugated with fluorescein isothiocyanate. Representative images are shown. (**D**) Cross-sectional area was quantitatively estimated. Data were shown as mean ± SD (n = 4 for Runx2^fl/fl^ mice, Runx2 CKO mice).

To examine whether Runx2^+^ myeloid cells influenced vascular function after MI, we assessed capillary density in post-infarct myocardium 14 days after MI using immunohistochemical staining with anti-CD31 antibody (Fig. 4A,B). Though there was no difference in capillary density before MI between control and Runx2-deficient mice (data not shown), capillary density was decreased in Runx2 CKO mice compared with control mice. Moreover, there were no differences in cardiomyocyte surface area between control and Runx2 CKO mice after MI (Fig. 4C,D), proposing that the decrease of capillary density in Runx2 CKO mice was mainly derived from impaired angiogenesis, but not from myocyte hypertrophy.

**Figure 5:**
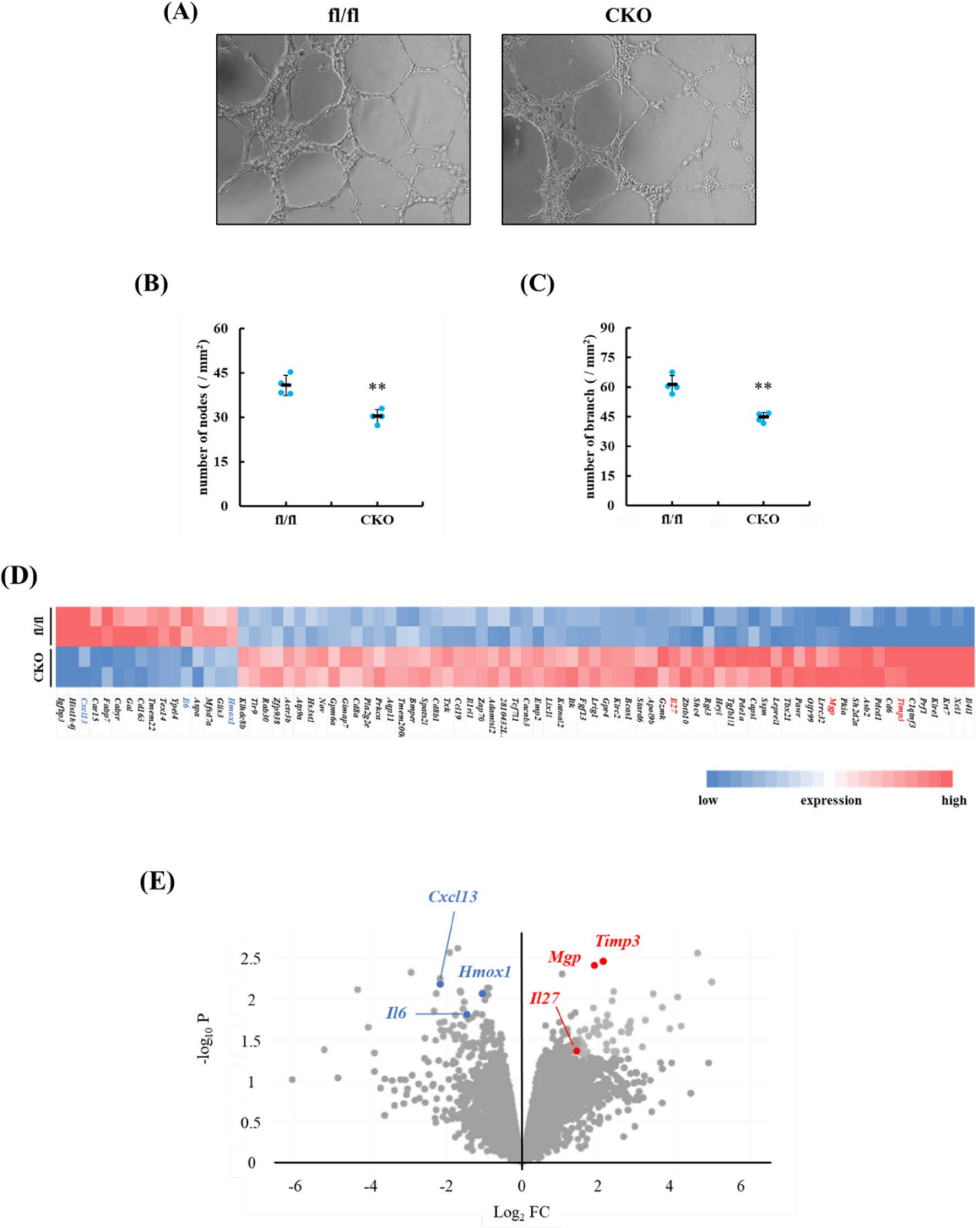
Runx2 ablation impaired angiogenic activity of infiltrated myeloid cells following MI. (**A**–**C**) CD11b^+^ cells were prepared from Runx2^fl/fl^ and Runx2 CKO hearts 7 days after MI and cultured for 24 h. Conditioned media from CD11b + myeloid cells of Runx2^fl/fl^ and Runx2 CKO were added to HUVECs. After 8 h, tube formation of HUVECs was evaluated. (**A**) Representative images of tube formation are shown. The number of nodes (**B**) and branches (**C**) was measured. Data are shown as mean ± SD (n = 4 for each condition) *P < 0.05 vs Runx2^fl/fl^ by student’s *t*-test. (**D**, **E**) CD11b^+^ F4/80^+^ macrophages were isolated from hearts of Runx2^fl/fl^ and Runx2 CKO mice at 7 days after MI. Total mRNA was extracted from the cells and RNA-sequence was performed (n = 2 in each group). Pro-angiogenic factors were indicated by blue letters, while anti-angiogenic factors by red (**D**) Heat map shows the gene with > 2.0-fold change between Runx2^fl//fl^ and Runx2 CKO with a *P*-value of < 0.05. (**E**) Volcano plots represent angiogenic gene expression compared Runx2^fl/fl^ vs Runx2 CKO.

To address the involvement of Runx2^+^ myeloid cells in angiogenesis, we analyzed the angiogenic activities of CD11b^+^ cells. CD11b^+^ cells were prepared from post-infarct hearts of control and Runx2 CKO mice and cultured for 24 h. HUVECs were cultured in conditioned media from CD11b ^+^ cells from control or Runx2 CKO mice that were exposed to MI. The tube formation assays demonstrated that the conditioned media from Runx2-depleted CD11b^+^ cells exhibited low angiogenic activity, compared with those from control myeloid cell culture (Fig. 5A–C). To address the mechanisms by which Runx2-expressing myeloid cells induced angiogenesis, RNA-sequence analysis was performed using sorted CD11b^+^ F4/80^+^ cells (Fig. 5D,E), and revealed that there was clear difference in the transcriptional profile between Runx2^fl/fl^ and Runx2 CKO macrophages. Interestingly, the expression of pro-angiogenic gene, *Cxcl13*^21^, *Il-6*^22^ and *Hmox1*^23^ was down-regulated, but that of anti-angiogenic gene, *Timp3*^24^, *Mgp*^25^ and *Il-27*^26^ was up-regulated in Runx2-dificient macrophages. These data suggest that Runx2^+^ myeloid cells could ameliorate post-infarct cardiac remodeling, at least partially, by regulating vascular function.

## Discussion

Myeloid cells play important roles in cardiac remodeling after myocardial injury^27,28^. In the present study, we demonstrated that the expression of Runx2 was upregulated in the post-infarct myocardium. Runx2 was mainly expressed in myeloid cells that infiltrated into the border zone. Myeloid cell-specific deletion of *Runx2* gene exacerbated cardiac remodeling after MI, associated with the impaired capillary formation. Consistently, the myeloid cells from the infarct myocardium of Runx2-deficient mice exhibited reduced angiogenic activity, compared with control mice, analyzed by tube formation assay in vitro.

Recent advances in osteoimmunology have proposed the concept that bone-related genes are closely associated with immune/inflammatory reactions^29^. However, it remains to be clarified whether Runx2, a master regulator of osteoblast differentiation^18^, regulates inflammatory reactions. In cardiovascular diseases, the pathological roles of Runx2 have been reported. For example, the ectopic expression of Runx2 in vascular smooth muscle cells results in vascular calcification in the atherosclerosis^19^. Here, we demonstrated that Runx2^+^ myeloid cells infiltrated into post-infarct myocardium and that Runx2 in myeloid cells prevented adverse cardiac remodeling after MI, providing the evidence for the physiological significance of Runx2 as a novel repair/regenerative signaling.

The myocardial expression of Runx2 was upregulated 4–7 days after MI, when macrophages are main infiltrating myeloid cells in post-infarct myocardium. Macrophages are classically classified into 2 subsets, M1 and M2^30^, and have been considered to have respective roles, such as inflammatory and anti-inflammatory effects. However, it was demonstrated that both M1 and M2 macrophages promote angiogenesis^31^. In this context, it is interesting that Runx2 was expressed both in M1 and M2 population of macrophages, because myeloid cells from post-infarct Runx2 CKO hearts showed reduced angiogenic activity. Thus, these data propose that the myeloid expression of Runx2 might explain the angiogenic property of M1 and M2 macrophages, as a common feature of these 2 subsets, though further experiments would be needed using M1- and/or M2-specific Runx2 CKO mice.

In Runx2 CKO hearts, capillary density was reduced after MI. Angiogenesis is a critical process of tissue repair after injury and impaired vascular function exacerbates adverse cardiac remodeling, including ventricular rupture^32,33^. Consistent with the in vivo results, conditioned media from Runx2-null CD11b^+^ cells failed to promote endothelial tube formation in comparison with control CD11b^+^ cells, suggesting that Runx2 in myeloid cells regulates post-infarct angiogenesis. To address angiogenesis-related factors responsible for Runx2-mediated regulation of angiogenesis in post-infarct myocardium, we performed RNA-sequence analysis using CD11b^+^ F4/80^+^ cells that infiltrated into the hearts after MI and found that the production of pro-angiogenic factors, such as IL-6, CXCL13, and Hmox1, was reduced in Runx2-null CD11b^+^ F4/80^+^ cells, while that of some anti-angiogenic factors, such as Mgp1, Timp3, and IL-27, was increased, proposing the hypothesis that the combined effects of reduced pro-angiogenic and enhanced anti-angiogenic activities resulted in the impaired angiogenesis. Thus, it could be suggested that Runx2^+^ myeloid cells could ameliorate post-infarct cardiac remodeling, at least partially, by regulating vascular functions.

This study has some limitations. First, we exclusively used male mice throughout this study to avoid the effect of estrogen cycle. According to the previous report^34^, the biological function of macrophages may be dependent on sex difference during cardiac remodeling. Further studies are needed to determine the function of Runx2-expressing myeloid cell in female during cardiac remodeling. Another limitation is the hematological difference of peripheral blood at baseline. In the present study, lymphocytes were decreased in number in Runx2 CKO mice at baseline. Though further study would be required to elucidate how lymphocytes decreased in Runx2 CKO mice, it is unlikely that the post-infarct cardiac remodeling was exacerbated in Runx2 CKO mice due to the decrease in lymphocytes, because Rag1^-/-^ mice, which lack lymphocytes, had significantly smaller infarct size than control mice after MI^35^ unlike Runx2 CKO mice.

In conclusion, myeloid cell-specific ablation of *Runx2* gene exacerbated cardiac remodeling after MI, indicating that Runx2^+^ myeloid cells play important roles in cardioprotection. Thus, the modulation of Runx2^+^ cells could be a novel therapeutic strategy against HF.

## Methods

### Animals

All animal experiments were carried out under the guidelines set by the Institutional Animal Care and Use Committees at Osaka University and RIKEN Kobe Branch. All the animal experiments in this study conformed to the Guide for the Care and Use of Laboratory Animals, Eighth Edition, updated by the US National Research Council Committee in 2011 and The Animal Research: Reporting of In Vivo Experiments (ARRIVE) guidelines, and were approved by Animal Experimentation Committee of Osaka University and Institutional Animal Care and Use Committee of RIKEN Kobe Branch. At the endpoints of all experiments, mice were anesthetized by inhalation of an overdose of isoflurane and the hearts were harvested. All efforts were made to minimize suffering.

### Generation of myeloid cell-specific Runx2 deficient mice

Based on the previous report^36^, the targeting vector with loxP sites flanking exon3 in the mouse *Runx2* gene was constructed to generate Runx2-floxed mice (Accession No. CDB0970K: http://www2.clst.riken.jp/arg/mutant%20mice%20list.html). Heterozygous Runx2-floxed mice were crossed with transgenic mice expressing LysM-Cre recombinase, generously gifted by Dr. Kiyoshi Takeda, Osaka University. The obtained heterozygous knockout mice were crossed with each other to develop homozygous Runx2-deficient (Runx2 CKO) mice. Homozygous Runx2-floxed mice (fl/fl) were used as control. The primers used for genotyping of Runx2-floxed mice are shown as follows: Runx2 forward, 5’-GTCGTCAGACCGAGAAGTGG-3’; reverse, 5’-GAAGTTAACAGCTTGCAGTAG-3’. The PCR reaction produces 613 bp band for WT allele and 819 bp band for the floxed allele.

### Counting leukocyte fraction

Peripheral blood was harvested from Runx2^fl/fl^ and Runx2 CKO mice at baseline. For inhibition of coagulation, EDTA-2 K (FUJIFILM, 340–01511) was added at a final concentration of 1.5 mg/mL. The analysis was contracted to BioSafety Research Center Inc (Japan).

### Myocardial infarction model

Myocardial infarction (MI) was generated by coronary artery ligation in C57BL/6 male mice (Japan SLC) and Runx2 CKO male mice (8–14-week-old) as previously described^37^.

### Quantitative RT-PCR

Total RNA was prepared from hearts at various time points after coronary ligation. Total RNA (1 μg) was subjected to first-strand cDNA synthesis using the oligo-deoxythymidine standard primer. The expression of *Runx2, Collagen1a1, Collagen3a1, Tgfβ1* and glyceraldehyde 3-phosphate dehydrogenase (*Gapdh*) gene was quantified by quantitative RT-PCR using the SYBR Green kit (Applied Biosystems, 4385612). The primers used in this study are shown as follows: mouse *Runx2* forward, 5’-CATCACCATCTTCCAGGAGCG-3’; reverse, 5’-GAGGGGCCATCCACAGTCTTC-3’, mouse *Gapdh* forward, 5’-CGGGAATGATGAGAACTACTC-3’; reverse, 5’-GTGAAACTCTTGCCTCGTCCG-3’, mouse *Collagen1a1* forward, 5’-GCGAGTGCTGTGCTTTCTG-3’; reverse, 5’-TCCCTCGACTCCTACATCTTC-3’, mouse *Collagen3a1* forward, 5’-ATGCCCACAGCCTTCTACAC-3’; reverse, 5’-CCCAGGGTCACCATTTCTCC-3’, mouse *Tgfβ1* forward, 5’-CTCCCGTGGCTTCTAGTGC-3’; reverse, 5’-GCCTTAGTTTGGACAGGATCTG-3’.

### Immunoblotting

Proteins from MI hearts or infiltrating cells were separated by SDS-PAGE and transferred to polyvinylidene difluoride membrane (Merck Millipore, IPVH00010). The membrane was blocked with 2% skim milk for 1 h, followed by incubation with rabbit anti-Runx2 (Cell Signaling Technology, #12556, 1/1000) and mouse anti-GAPDH (Merck Millipore, #MAB374, 1/1000) antibodies overnight at 4 °C. After primary antibody reaction, membranes were incubated with HRP-conjugated anti rabbit IgG (Cell Signaling Technology, #7074, 1/2000) or HRP-conjugated anti mouse IgG (Jackson ImmunoResearch, 115–035-062, 1/2000) for 1 h at room temperature. Chemiluminescence reagent, Chemi-lumi one super, (Nacalai tesque, 02230–30) were used for detection, and bands were developed using a LAS4010 Imaging system (Cytiva).

### Histological estimation of cardiac fibrosis

Cardiac fibrosis was histologically estimated 14 days after coronary ligation. The frozen sections (5-μm-thick) were prepared from the portion ~ 400 μm distal to the ligation point and stained with Masson's trichrome. Photomicrographs were taken, and the areas were measured using Image J (National Institutes of Health). The fibrotic area was calculated as a percentage of LV area by a researcher who was blinded to the experimental

### Immunofluorescent microscopic analyses

Frozen sections (5-μm-thick) were prepared 7 days after coronary ligation. The sections were fixed with 4% paraformaldehyde (PFA) and blocked with 3% bovine serum albumin (BSA) in Tris-buffered saline containing 0.1% Triton X-100. Then, the sections were stained with rabbit anti-Runx2 (Cell Signaling Technology, #12556, 1/100), goat anti-periostin (Santa Cruz Biotechnology, sc-49480, 1/100) and rat anti-CD68 (Invitrogen, #14-0681-80, 1/100) antibodies overnight at 4 °C. Alexa Fluor 488-conjugated goat anti-rat IgG (Invitrogen, A-11006, 1/200), Alexa Fluor 488-conjugated donkey anti-goat IgG (Invitrogen, A-11055, 1/200) and Alexa Fluor 546-conjugated donkey anti-rabbit IgG (Invitrogen, A-10040, 1/200) were used as secondary antibodies. Nuclei were also stained with Hoechst 33258. The sections were examined using a fluorescent microscopy system (KEYENCE, BZ-X710).

### Immunohistochemical analyses

The frozen sections and paraffin-embedded sections were prepared from the hearts. Capillary density and Runx2 expression were examined by immunohistochemical staining using the Vectastain ABC kit (Vector Laboratories) with rat anti-CD31 antibody (BD Biosciences, 550274, 1/100) and rabbit anti-Runx2 antibody (Cell signaling Technology, #12556, 1/100). Immunostained sections were examined under a light microscope (KEYENCE, BZ-X710). The number of CD31^+^ cells at the border zone was quantitatively estimated by a researcher blinded to the assay conditions.

### TUNEL staining

Frozen sections (5-μm-thick) were prepared 7 days after MI. The sections were fixed with 4% PFA and blocked with 3% bovine serum albumin (BSA) in Tris-buffered saline containing 0.1% Triton X-100. The sections were treated as instructed in the protocol accompanying in situ Apoptosis Detection Kit (Takara, MK500). Then, the sections were stained with goat anti-cardiac Troponin-I (Abcam, ab56357, 1/100) antibody at room temperature for 1 h. Alexa Fluor 546-conjugated donkey anti-goat IgG (Invitrogen, #A-11056, 1/200) was used as a secondary antibody. Nuclei were also stained with Hoechst 33258. The sections were examined using a fluorescent microscopy system (KEYENCE, BZ-X710).

### Determinations of cell surface areas

Heart sections were fixed with 4% PFA and incubated with wheat germ lectin conjugated with fluorescein isothiocyanate for 1 h at room temperature. Cell surface areas of 100–200 cardiomyocytes in 30–50 fields were measured in each group by a researcher blinded to the assay conditions.

### Preparation of CD11b^+^ cells by Magnetic activated cell sorting system

Infarcted hearts were harvested and washed with PBS, perfused with 0.025% collagenase solution [0.0125% collagenase type B (Roche), 0.0125% collagenase type D (Roche), and 0.002% collagenase type XIV (Sigma-Aldrich)] and minced into pieces. The pieces of heart tissue were digested with 0.1% collagenase solution [0.05% collagenase type B (Roche), 0.05% collagenase type D (Roche), and 0.002% collagenase type XIV (Sigma-Aldrich)] at 37 °C. Then the cells were filtered through a 70 μm cell strainer and suspended in PBS / 3% fetal bovine serum (FBS). Erythrocytes were removed from the cells using Lysing buffer (BD Biosciences, 555899). After centrifugation, the pellet was resuspended in 40 / 80% Percoll buffer layers (Cytiva, 17-0891-02) and the leukocyte-rich fraction was collected from the interface between 40 and 80% Percoll buffer by 25-min-centrifugation, followed by the blockade of Fc receptors. Biotinylated anti-CD11b antibody (BioLegend, 101203) was added to cell suspension (1 µl / 1 × 10^6^ cells) and incubated for 30 min on ice. After centrifugation, the cells were resuspended in BD IMag Buffer (BD Biosciences, 552362), Streptavidin Particles Plus-DM (BD Biosciences, 557812) was added five times the amount of biotin anti-CD11b antibody, then incubated for 30 min on ice. The column of the cell suspension was attached to the magnet for 8 min and cell population attached to the wall of the column was collected in PBS / 3% FBS so that the “positive” fraction could be collected. Consistent with previous reporte^38^, Percoll and MACS cell separation enriched leukocytes to a high purity of 98%.

### Flow cytometric analysis

Single cell suspensions were isolated from hearts 4 or 7 days after MI according to Percoll method. The cells were filtered through a 70 μm cell strainer and incubated with 1 μL / 1.0 × 10^6^ cells rat anti-CD11b-APC/Cy7 (BioLegend, 101225), rat anti-CD45-FITC (BioLegend, 103107), rat anti-F4/80-PE (BioLegend 123109), rat anti-CD11b-PE (Biolegend,101207), rat anti-CD86-APC (BioLegend, 105007), rat anti-CD206-APC (BioLegend, 141707), and rabbit anti-Runx2 (Described above) antibodies on ice for 1 h after Fc receptor blockade by incubation with 1 μL / 1.0 × 10^6^ cells rat anti-CD16/32 (BioLegend, 101301) antibody on ice for 30 min. FITC Rat IgG2a, κ Isotype Ctrl antibody (BioLegend, 400505), PE Rat IgG2a, κ Isotype Ctrl antibody (BioLegend, 400507), APC Rat IgG2a, κ Isotype Ctrl antibody (BioLegend, 400511), and APC/Cy7 Rat IgG2a, κ Isotype Ctrl antibody (BioLegend, 400523) were used for negative control antibodies. Intracellular Runx2 labeling was performed using FoxP3/Transcription Factor Staining Buffer set (Invitrogen, 00-5523-00) according to manufacturer’s protocol. Flow cytometric analysis was performed with FACS AriaII (BD Biosciences) and data were analyzed using FlowJo software (Tree Star).

### Gravimetric study

The hearts and lungs were harvested and washed in phosphate buffered saline (PBS). The weight of these organs was measured using an electronic balance and normalized to the tibia length or body weight.

### Analysis of cardiac function

Two-dimensional and motion mode (M-mode) transthoracic echocardiography was performed using an iE33 model equipped with a 15-MHz transducer (Philips). Echocardiographic measurements were taken in M-mode. Left ventricular internal diameters at end-diastole (LVIDd), left ventricular internal diameters at end-systole (LVIDs), and fractional shortening (FS) were calculated with the established standard equation. The investigator was blinded to the identity of the mice for analysis.

### Cell culture

BMDMs were isolated from femurs of adult Runx2^fl/fl^ or Runx2 CKO mice and differentiated into macrophages using RPMI1640 supplemented with 20 mM recombinant mouse M-CSF (R&D systems, 416-ML-010) containing 10% FBS and 1% penicillin-streptomycin at 37 °C, 5% CO_2_ for 7 days. BMDMs (5.0 × 10^5^ cells/mL) were seeded 6-well culture plates. BMDMs were stimulated with 100 ng/mL LPS (Sigma-Aldrich, O111:B4) for 24 h, and then, total RNA was extracted.

### Tube formation assay

1.0 × 10^4^ human umbilical vein endothelial cells (HUVEC) in 50 µL 2% FBS/EGM2 basal medium (Lonza, CC-3156) were seeded into 96-well plates which were treated 50 µL extracellular matrix solution (Abcam, ab204726) for 1 h at 37 °C. After 8 h incubation with conditioned media, the cells were evaluated for a number of nodes and branches.

### RNA-sequencing analysis

Total RNA was prepared from sorted CD11b^+^ F4/80^+^ macrophages from post-infarct myocardium (2–5 × 10^4^ cells/each population) using the Qiazol (QIAGEN) and miRNeasy Mini Kit (QIAGEN). cDNA was generated using a SMART-Seq HT Kit (Clontech). Each library was prepared using Nextera XT (Illumina) according to the manufacturer’s instructions. Sequencing was performed on an Illumina HiSeq 2500 platform in a 75-base single-end mode. Illumina Casaval.8.2 software was used for basecalling. Sequenced reads were mapped to the mouse reference genome sequence (mm10) using TopHat ver.2.0.13 in combination with Bowtie2 ver.2.2.3 and SAMtools ver.0.1.19. The fragments per kilobase of exon per million mapped fragments (FPKMs) was calculated using Cuffnorm ver.2.2.1. Volcano plots were created using Biojupeis^39^.

### Statistical analysis

Data are presented as mean ± SD. The χ^2^ goodness of fit test was first used to determine data normality, and the F test was used to determine equal variance. If the data passed the evaluation of normality, statistical significance was determined by Student’s or Welch’s *t*-test for 2 groups, and One-way analysis of variance (ANOVA) with the Dunnett test for multiple comparisons. The nonparametric tests, such as Kruskal-Wallis test, steels test, or Mann-Whitney *U* test were used when the data displayed abnormal distribution. Survival curves were obtained by the Kaplan-Meier method and compared by the log-rank test. Differences were considered statistically significant when the calculated (two-tailed) *P*-value was < 0.05.

## Supplementary Information

Supplementary Information.

## Data Availability

The data that support the findings of this study are available from the corresponding authors, Y. F., upon reasonable request.
