# Supplementary material for: Myeloid cell-specific ablation of Runx2 gene exacerbates post-infarct cardiac remodeling
Source: Sci Rep. 2022 Oct 5;12:16656. doi: 10.1038/s41598-022-21202-7 (PMC9534857; doi:10.1038/s41598-022-21202-7)
Supplement: Supplementary file 1 — Supplementary Information. [file 41598_2022_21202_MOESM1_ESM.docx]

**
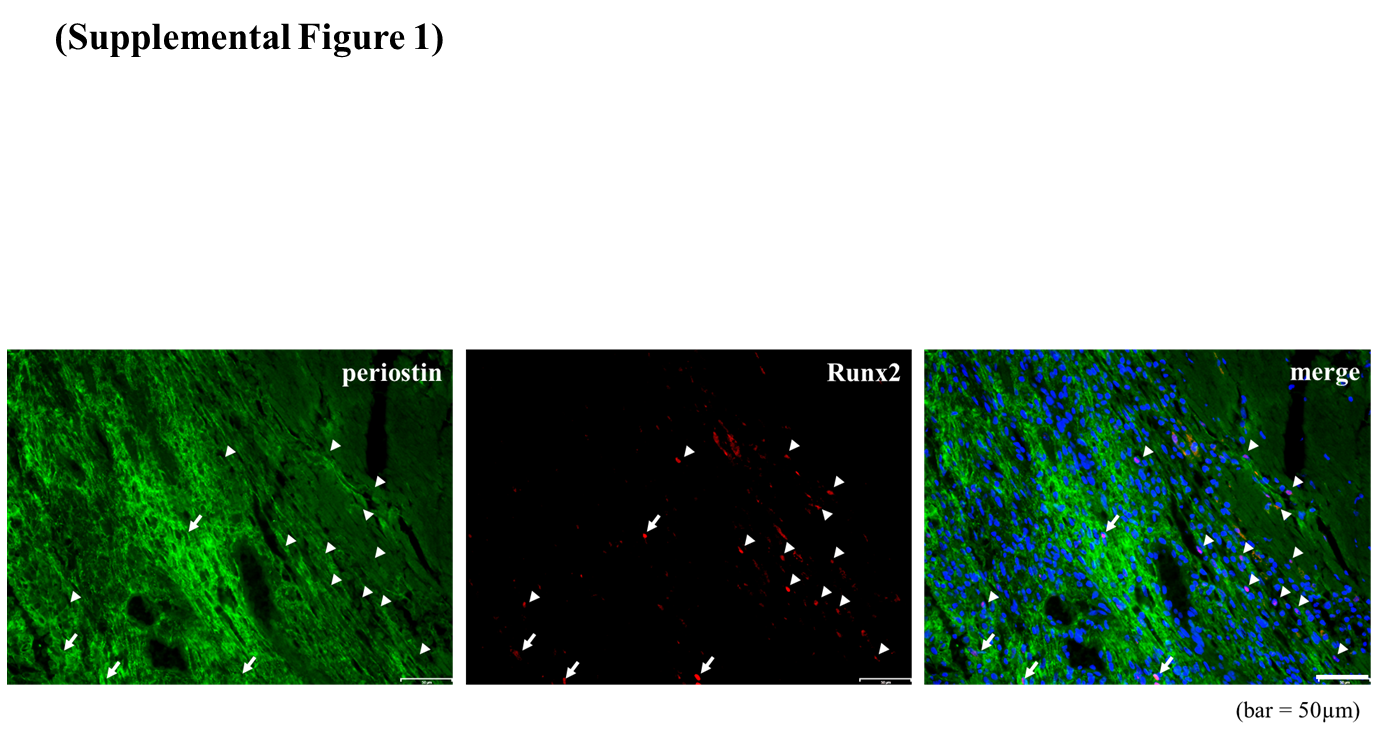
**

**Supplemental Figure S1. *Runx2 was expressed in myofibroblasts.***

Paraffin-embedded sections of infarcted myocardium were prepared from the heart 7days after MI. The sections were stained anti-periostin, myofibroblast marker, and anti-Runx2 antibodies. Representative images are shown. Arrow, periostin^+^ Runx2^+^ cells, Arrow heads, periostin^-^ Runx2^+^ cells (bar=50µm)

**
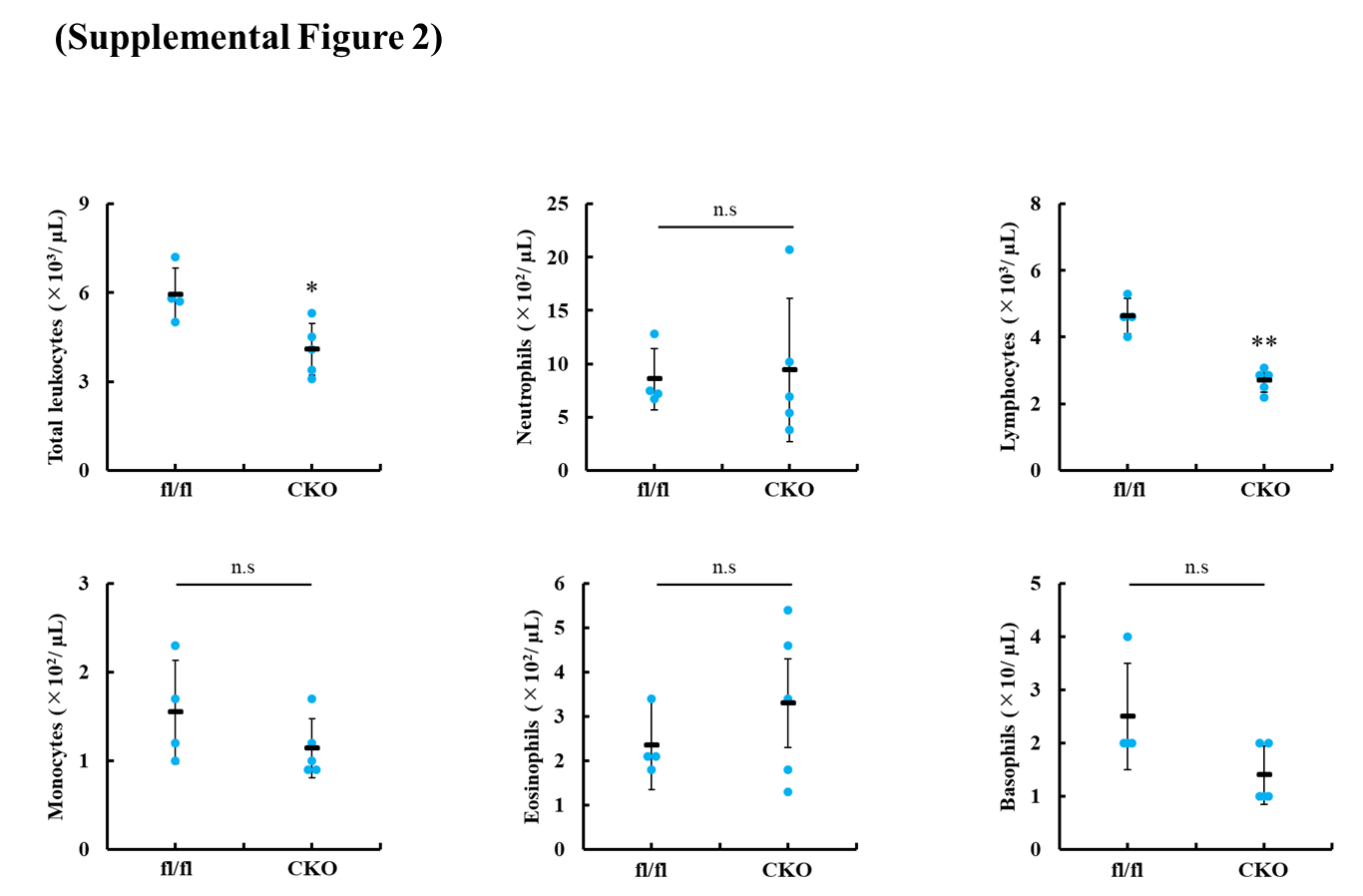
**

**Supplemental Figure S2. *Myeloid cell-specific Runx2 ablation decreased lymphocytes in number.***

Peripheral blood was collected for Runx2^fl/fl^ and Runx2 CKO mice at steady condition. Each leukocyte fraction in blood was calculated (n=4 for Runx2^fl/fl^ mice, n=5 for Runx2 CKO mice). Data are shown mean ± SD. **P*<0.05 vs Runx2^fl/fl^ by Student’s *t*-test.


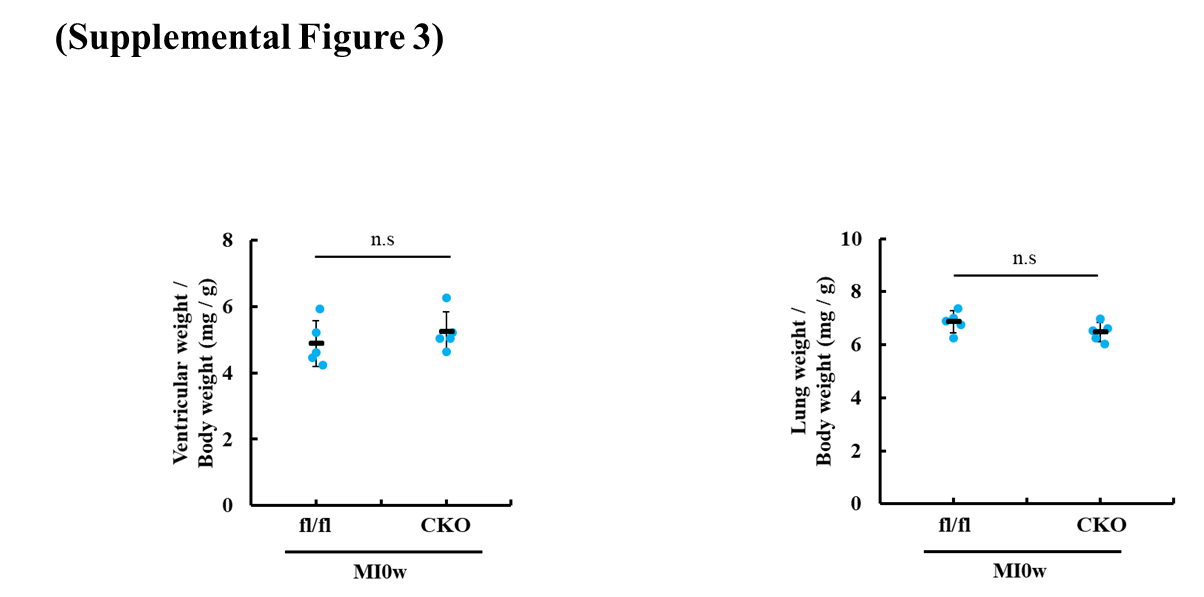


**Supplemental Figure S3. *Myeloid cell-specific Runx2 ablation didn’t affect ventricular and lung weight at baseline.***

Ratios of ventricular weight to body weight and lung weight to body weight were calculated at baseline condition. Data were shown as mean ± SD (n=5), n.s indicates not significant.

**
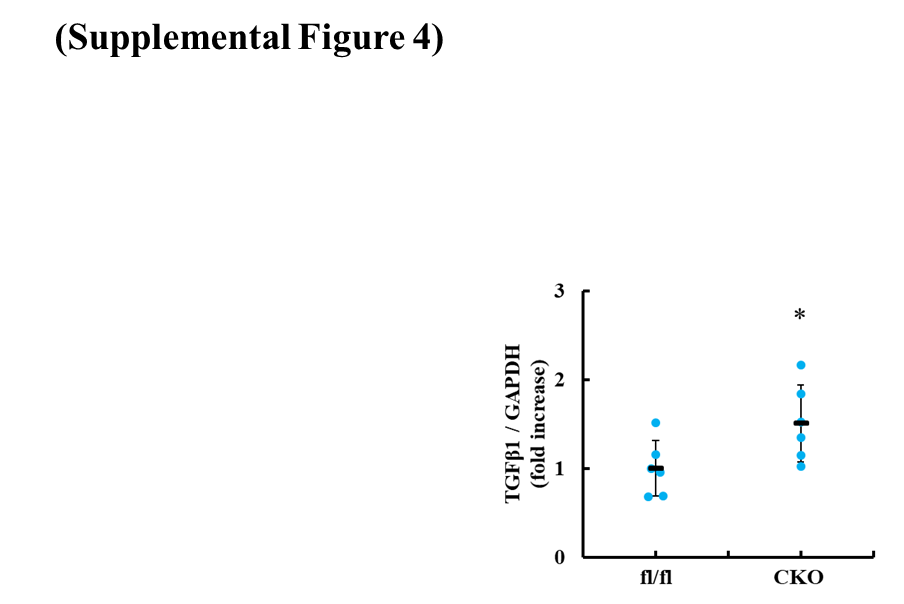
**

**Supplemental Figure S4. *Runx2 ablation induced Tgfβ1 expression in bone marrow-derived macrophages (BMDMs).***

BMDMs were stimulated with LPS at 100 ng/mL for 24 hours. The expression of *Tgfβ1* mRNA was examined using quantitative RT-PCR. The results were normalized to that of GAPDH. Data are shown mean ± SD (n=6 for Runx2^fl/fl^ and Runx2 CKO mice). **P*<0.05 vs Runx2^fl/fl^ by Student’s *t*-test.

**
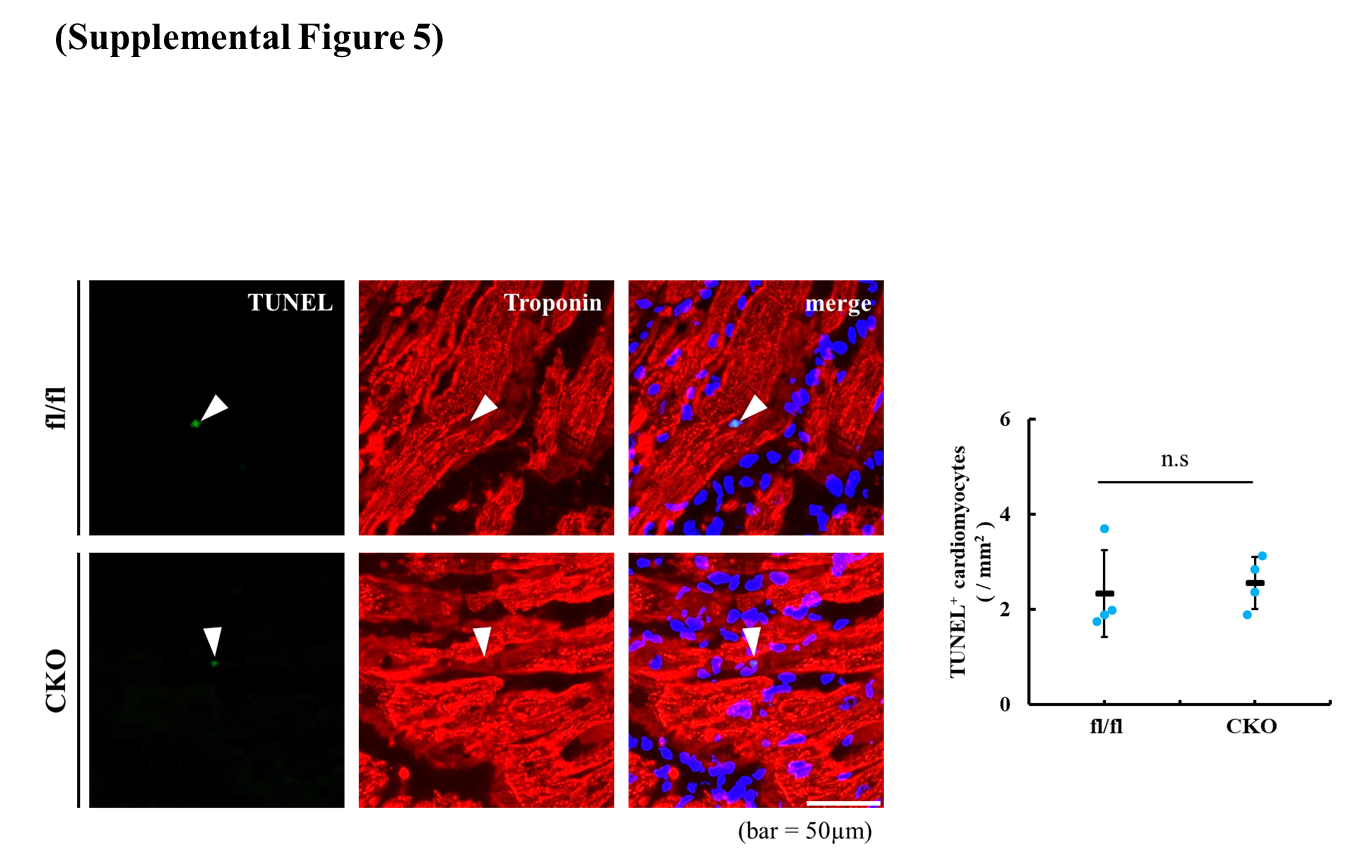
**

**Supplemental Figure S5. *Runx2 ablation in myeloid cells didn’t affect cardiomyocytes apoptosis after MI.***

Frozen sections were prepared from the hearts 7 days after MI. The sections were co-stained with TUNEL and anti-Troponin I antibody, cardiomyocyte marker. Representative images are shown. Arrow heads: TUNEL^+^ cardiomyocytes. n.s indicates not significant.

**
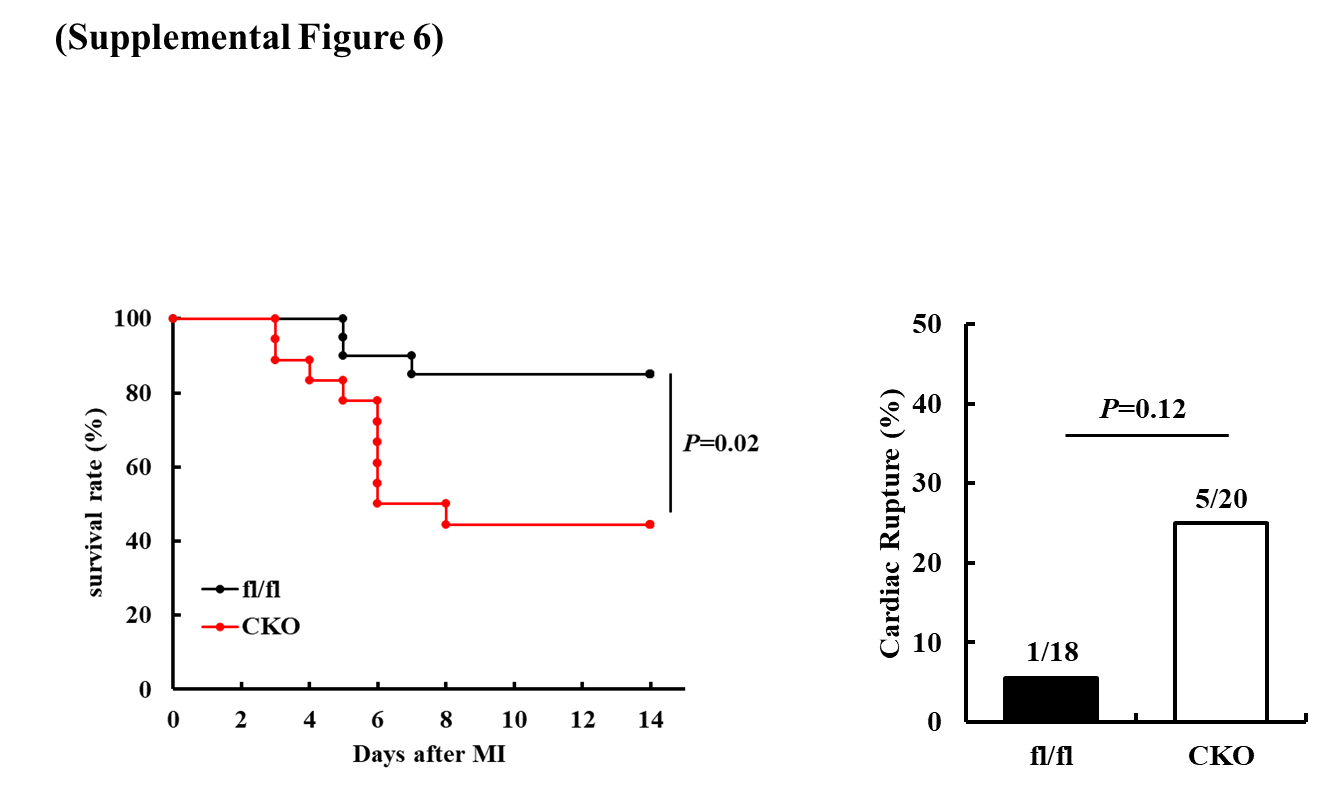
**

**Supplemental Figure S6. *Survival rate was reduced in Runx2 CKO mice after MI.***

(Left) Kaplan-Meier survival curve indicated the survival rate of Runx2^fl/fl^ and Runx2 CKO mice following MI (n = 18 for Runx2^fl/fl^ mice, n = 20 for Runx2 CKO mice). ***P*<0.01 vs Runx2^fl/fl^ by Kaplan-Meier log-rank test.

(Right) Incidence of cardiac rupture in Runx2^fl/fl^ and Runx2 CKO mice at 14 days after MI. *P*-value was calculated by Fisher’s exact probability test.
